# How to Indicate Implantable Cardioverter‐Defibrillator in Patients With Malignancy

**DOI:** 10.1002/joa3.70148

**Published:** 2025-07-16

**Authors:** Naoya Kataoka, Teruhiko Imamura

**Affiliations:** ^1^ Second Department of Internal Medicine University of Toyama Toyama Japan

**Keywords:** amiodarone, cardiomyopathy, ventricular tachyarrhythmia

To Editor,

The indication for cardiac implantable electronic devices in patients with malignancy remains a matter of ongoing debate. Current guidelines generally advocate for device implantation only in patients with an anticipated survival exceeding 1 year. Nevertheless, the authors report that implantable cardioverter‐defibrillator (ICD) therapy conferred superior long‐term survival compared to amiodarone in oncology patients, including those with advanced‐stage cancer [1]. This finding raises several important concerns.

In Japan, prevailing guidelines explicitly discourage ICD implantation in patients whose life expectancy is less than 1 year, designating it as a Class III indication [2]. Accordingly, within the context of this Japan‐based study, ICDs should have only been implanted in patients with an estimated survival beyond 1 year [1]. It is important to clarify whether patients with an expected life expectancy of < 1 year were included in the study. In contrast, amiodarone prescription is not governed by such stringent prognostic thresholds. Consequently, the ICD cohort may have inherently comprised patients with more favorable baseline prognoses compared to those receiving amiodarone. It would be informative to clarify whether ICD intervention effectively averted sudden cardiac death in this population.

The therapeutic intent behind amiodarone administration in this study also warrants further elucidation. Notably, only 16.7% of patients in the amiodarone group had a documented history of ventricular arrhythmias [1]. It is likely that the remaining individuals were treated with amiodarone for rate or rhythm control in the context of supraventricular tachyarrhythmias [3]. Such arrhythmias are frequently refractory in patients with systemic deterioration, suggesting that amiodarone may have been preferentially used in individuals with more advanced or decompensated clinical status.

An emerging area of interest within the field of onco‐cardiology involves cardiovascular toxicities associated with anticancer agents, most notably immune checkpoint inhibitors, anthracyclines, and fluoropyrimidines, including the development of cardiomyopathy [4]. The prophylactic utility of ICDs in this specific patient subset remains unclear. In the present study, 62% of ICD recipients underwent implantation for secondary prevention [1]. Among these patients, how many had a history of anticancer drug therapy?

## Ethics Statement

The authors have nothing to report.

## Consent

The authors have nothing to report.

## Conflicts of Interest

The authors declare no conflicts of interest.

## Linked Articles

H. Kida, T. Morishima, E. Uza, et al., “Prognostic Comparison Between Implantable Cardioverter‐Defibrillator and Amiodarone in Cancer Patients,” *Journal of Arrhythmia* 41 (2025): e70093, https://doi.org/10.1002/joa3.70093.

## Data Availability

The authors have nothing to report.
